# Listening to vaccine refusers

**DOI:** 10.1007/s11019-021-10055-y

**Published:** 2021-10-30

**Authors:** Kaisa Kärki

**Affiliations:** Practical Philosophy, University of Helsinki, PL 24, 00014 Helsinki, Finland

**Keywords:** Vaccine refusal, Herd immunity, COVID-19, Exit, Free riding

## Abstract

In bioethics vaccine refusal is often discussed as an instance of free riding on the herd immunity of an infectious disease. However, the social science of vaccine refusal suggests that the reasoning behind refusal to vaccinate more often stems from previous negative experiences in healthcare practice as well as deeply felt distrust of healthcare institutions. Moreover, vaccine refusal often acts like an exit mechanism. Whilst free riding is often met with sanctions, exit, according to Albert Hirschman’s theory of exit and voice is most efficiently met by addressing concerns and increasing the quality and number of feedback channels. If the legitimate grievances responsible for vaccine refusal are not heard or addressed by healthcare policy, further polarization of attitudes to vaccines is likely to ensue. Thus, there is a need in the bioethics of vaccine refusal to understand the diverse ethical questions of this inflammable issue in addition to those of individual responsibility to vaccinate.

## Introduction

Vaccine refusal in bioethics is often perceived as a ‘collective action problem’ in which individual citizens are free riding on the herd immunity of others (e.g., Giublini 2020; Savulescu 2021; Browne 2016). According to Gareth Cullity (1995), free riding is a situation in which agents optimize their own interest by not paying for the public goods they consume. Free riding is deemed to be wrong because it includes objectionably preferential treatment of oneself over others.

Bioethical discussion on vaccine refusal has centered on questions of individual obligation to vaccinate. At the same time there is a growing need to address vaccine refusal in health care policy in the most effective way. When facing the COVID-19 crisis, vaccination programs need to be able to address all citizens. For example, between one third and one fifth of American adults plan to refuse a COVID-19 vaccine (Motta 2021) even though herd immunity to Covid-19 requires vaccine coverage of above 80%. It is currently an open question which policies are the most effective in influencing vaccine refusers (e.g., Dubé et al. 2015).

This article calls for a change of perspectives to the bioethics discussion on vaccine refusal. I argue that treating vaccine refusal as an instances of free riding, even though it brings out ethically central aspects about them, does not necessarily lead to effective interventions. This is because vaccine refusal is rarely consciously intended to be an instance of free riding on herd immunity; instead, it may mirror an attempt to exit a public good instead of free riding on one. If it is seen as exit mechanisms of a kind, vaccine refusal is better met by listening to the actual grievances of patients in healthcare thus preventing further polarization of attitudes about vaccines. Considering the social science of vaccine refusal demands that bioethicists look at this complex socio-political issue from a more diverse point of view than that of an individual obligation not to free ride. For instance, the injustices marginalized groups encounter in healthcare practice may explain motivations to refuse vaccines and in order to effectively manage them, factors leading to distrust should be addressed instead of only ascribing blame of free riding on herd immunity to individuals who do not experience the healthcare system as their own.

In the first part of the paper, I will show that based on empirical research on the reasoning behind vaccine refusal, free riding on herd immunity does not arise as a significant explanation of the motivations for this complex phenomenon.^1^ Then, I will demonstrate the usefulness of Hirschman’s theory of exit and voice for understanding the inflamed relationship between citizens and healthcare institutions in vaccine refusal. In the third part, I will show how both the exit approach and the social science of vaccine refusal leads to similar findings about how to effectively solve this issue. These findings emphasize respectful discussion with vaccine-hesitant patients instead of immediately ascribing blame to those who refuse to be vaccinated or display hesitancy about a vaccine. The concluding sections bring the discussion back to the change of perspectives needed in bioethics due to the urgency of tackling vaccine hesitancy. The World Health Organization had already flagged this as one of the ten most urgent threats to global health (WHO 2019) in 2019 before the Covid-19 crisis.

## Multiple grievances

Vaccine refusers are not a homogenous group (e.g., Asveld 2008). In their extensive review on research on the reasons for vaccine refusal, Yaqub et al. found that only one out of 38 articles cited conscious free riding as a reason for refusing vaccines (Yaqub et al. 2014). Conscious free riding on the herd immunity of others is only one rationale for vaccine refusal (Asveld 2008: 247). According to the current social science of vaccine refusal, its rationale partly stems from various grievances in healthcare practice.

Public worries about the safety and efficacy of vaccines explain part of vaccine refusal but others stem from other issues and perceived injustices in healthcare practice (Navin 2013). For instance, the sexist attitudes of physicians toward mothers and hostile responses of healthcare personnel to patient enquiries concerning vaccines arise from the empirical literature as reasons for vaccine refusal. Navin found that vaccine refusers have often been treated disrespectfully by physicians (2013: 247). Parents who refused vaccination for their children reported that pediatricians did not listen to them and did not offer adequate explanations on the safety and efficacy of vaccines, whereas the anti-vaccine figures were perceived as actually listening to the parents.

Ethnographical research on vaccine refusal suggests a similar line of reasoning. Poltorak et al. (2005) found that previous medical experiences with medical personnel influenced trust in vaccine recommendations. Helps et al. (2019) found that dismissive encounters with health professionals were significant factors behind vaccine refusal. Perceived dismissal of parental concerns caused distress and undermined trust to medical establishment (Helps et al. 2019: 5). Nurmi & Salmenniemi (2019) found that vaccine-hesitant parents had often experienced aggressive conduct in healthcare. For instance, they had been called “child killers” as a response to merely asking whether a vaccine was safe (Nurmi & Salmenniemi 2019: 66). In general, interactions with healthcare providers have been found to be the key in understanding how vaccine hesitancy emerges (Dubé et al. 2015: 4201).

Distrust of COVID-19 vaccines has been found to be most prevalent among individuals from ethnic minority groups, with lower annual income and of female gender (Paul et al. 2021; Callaghan et al. 2021). Ward et al. found that vaccine refusal is more prevalent among the poor and marginalized groups perhaps because they are more at risk of encountering difficult interactions with institutions or because of feeling completely abandoned by them (Ward et al. 2020). Perceived injustices in healthcare practice may explain why women, the poor and the marginalized are in danger of losing their trust in the medical establishment.

Vaccine refusal may also stem from distrust of the political establishment of the whole country, for instance, as happened in the case in the Nigerian boycott of polio vaccination (Larson et al. 2011). Yaqub et al. found that lack of public trust was a more cited reason for vaccine hesitancy than lack of knowledge (Yaqub et al. 2014: 3). Yaqub et al. found that, in general, healthcare workers are experiencing an increasing number of problems in gaining the trust of their patients (2014). This mistrust was found to be targeted at healthcare institutions—not only particular vaccines. In the Netherlands, for instance, the government was believed to be the target of extensive lobbying by vaccine-producing companies (Yaqub et al. 2014). In France, distance from the political system correlated with negative attitudes to COVID-19 vaccines (Ward et al. 2020). Those who felt close to radical parties, felt distance toward all parties, and did not vote during the last election were significantly more likely to refuse vaccines (Ward et al. 2020: 2).

Of course, it is possible that due to the social stigma, vaccine refusers are listing other motivations than free riding when asked. If one is to free ride on the herd immunity of others, it would be rational to make sure, or at least not interfere with, others who are getting vaccinated (Dare 1998: 144). Successful free riding is dependent on the complicity of others so that herd immunity is successfully formed in the first place. However, some vaccine refusers spread anti-vaccination material, which would be irrational if they were trying to free ride on herd immunity. Sometimes vaccine refusal does not seem to altogether meet the criteria for successful free riding on herd immunity.^2^

In conclusion, when enquiring about the motivations behind vaccine refusal, instead of consciously free riding on the herd immunity of others, the social science of vaccine refusal suggests that the reasoning behind vaccine refusal more often stems from previous negative experiences in healthcare practice as well as a deeply felt distrust toward healthcare institutions. Free riding motives are occasionally found in research on vaccine refusal, however, and it is possible that some vaccine-hesitant individuals are free riding consciously, because their decisions to postpone getting vaccinated may hinge on the complicity of others in taking the risk of potential side-effects on their own behalf.

## Exiting a public good

In Hirschman’s theory of exit and voice, whether citizens express dissatisfaction through the open expression of dissent, *voice*, or through the invisible mechanism of voting with their feet, *exit*, is dependent on the perceived features of an organization (Hirschman 1970). Exit is a situation in which the articulation of grievances is perceived to be somehow not worthy of effort. Exit is taken “in the light of the prospects for the effective use of voice” (Hirschman 1970: 37). Citizens who have lost trust in the public expression of dissent are more likely to prefer silent mechanisms of voting with their feet.

The social scientific literature on vaccine refusal points toward thinking of vaccine refusal as an exit mechanism of a kind. As discussed in the previous part, according to the current social science of vaccine refusal, its rationale often stems from various grievances in healthcare practice and as well as diminished trust to healthcare institutions. According to Navin, a mother who has faced epistemic injustices in healthcare may rationally resist her oppression by abandoning this oppressive relationship and by creating “new forms of social life” (Navin 2013: 253). Nurmi and Salmenniemi recount that vaccine-refusing parents stopped talking about their choices to healthcare personnel after negative experiences and the following lack of trust (Nurmi and Salmenniemi 2018). Helps et al. (2019, 4) found that vaccine refusing parents ‘opted out’, not only of vaccination but of other Western values they perceived as problematic. Partisan membership and a lack of trust in public institutions arising from the Covid-19 vaccine hesitancy literature support the same conclusion. Vaccine refusers do not seem to find an established outlet for their frustrations in healthcare.

Hirschman reads both exit and voice as signs that the firm, state, or organization has deteriorating performance. However, public goods cannot be exited without exiting the whole community that provides them (Hirschman 1970: 101). Hirschman defines public goods as “goods that are consumed by all the members of a community so that their consumption by one member does not detract from the consumption of another” (Hirschman 1970: 101). He mentions crime prevention and national defense as prime examples of public goods but includes all such results of public policies that are enjoyed by everyone such as international prestige, advanced literacy, and public health (Hirschman 1970: 101). Public goods cannot be exited, because even if one were to opt out of a public school, one cannot exit from enjoying the benefits of the public school system as a whole. Public goods are shared, and they are all around us – it is impossible not to enjoy their benefits or not be bothered about their declining quality (Fennell 2001).

Herd immunity is usually seen as a public good in bioethics (e.g., Selgelid 2009; Dawson 2009). It includes a benefit that is shared by all members of a population and its benefit is disproportionate, meaning that while its benefit happens at the population level, the possible harm is borne by individuals. Even if one citizen were to oppose herd immunity to polio, only living in a society that provides this benefit would make the citizen enjoy the public good of herd immunity to polio.

It is an interesting question whether public goods can be exited. Exit from *supporting* a public good, can be perceived at least. If non-compliance reaches a certain level, in the case of herd immunity, the *consumption* of the public good itself may itself be jeopardized. Vaccine refusal resembles an attempt to exit a public good, because a collective exit from herd immunity is conceivable even if one member cannot exit it alone by refusing to vaccinate. In a democratic society, it is a matter of public debate whether something is considered to be a public good. If citizens have lost trust in the effectivity of public outcries, they may turn to more invisible forms of opposing policies.

Vaccine refusal read as an attempt to exit a public good is a signal that something else altogether is not functioning properly in the healthcare system. This can happen without major changes in healthcare services themselves – when the role and expectations of how healthcare is arranged is changing rapidly.

Hirschman points out that people can react to a public good as if it were a private one (Hirschman 1970: 105). In a society dominated by private goods, such confusions can happen (Hirschman 1970: 105). In healthcare, treating a public good as if it was a private good may be connected to the postmodern perception of the patient as a consumer — making consumer-like choices in the healthcare setting (Kata 2012). Kata has argued that the anti-vaccine movement takes advantage of this postmodern medical paradigm by framing the refusal to be vaccinated as part of the patient’s choice (Kata 2012: 3784). According to Kata, the Internet has increased the personalization of healthcare, transforming it into an arena of shared decision making between the patient and the professional (2012: 3779). The Internet seems to be only one change agent in this area.^3^ Yaqub et al. point out that the rhetoric emphasizing patient choice may partly explain the lack of trust in healthcare workers (2014: 7). This trend, they think, is part of a larger shift that focuses on the patient’s right to informed choice. Poltorak et al. (2005) also found that vaccine refusal is part of a wider change in how people relate to the state. According to their findings, the public health framing that emphasizes the role of public goods has little resonance with the current paradigm that now guides vaccine decisions (Poltorak et al. 2005: 717). Su et al. are already calling vaccine refusers “end *users* of health technologies” (2020).

## Effective interventions

Another strength of applying Hirschman’s theory to understanding vaccine refusal in addition to explaining its motivations is that it can help in designing effective interventions for it. The plea for democratic decision making, the need for equal treatment of patients at healthcare practice and the growing lack of public trust in the medical establishment all point toward different interventions than framing vaccine refusal only from the perspective of individual responsibility to vaccinate.

Exit is a sign that communication between citizens and the state is not functioning properly. According to Hirschman, institutions that do not wish to encourage the exit option should provide as many effective channels for articulation of criticism as possible. Then, the inevitable grievances stemming from actual quality failures could be addressed. Not only does the number of channels matter, but it is also important that the organization is responsive to the criticisms made (Hirschman 1970). Once the usefulness of the voice mechanism for maintaining good performance has been recognized, institutions can be designed in a way that reduces the cost of voice or rewards it (Hirschman 1970: 42). In healthcare, reducing the cost of voice would mean increasing the number of reporting channels for offensive behavior by physicians and developing ways to increase democratic decision making between physicians and patients.

Major changes in ways in which medicine is practiced have been called for to counter vaccine refusal (Navin 2013: 253). Getting rid of testimonial injustices, such as not hearing out mothers talking about the health problems of their children, and epistemic vices, such as an authoritarian way of transferring information without explanation, are needed (Navin 2013: 254). If gender prejudices contribute to the disrespectful communication between pediatricians and mothers, increasing gender equality in healthcare practice would eventually reduce vaccine refusal.

According to public health communication studies, it has been deemed to be important to treat vaccine sceptics respectfully (Fahlquist 2018). A good starting point for a respectful discussion, according to Fahlquist, is taking the concerns of vaccine sceptics seriously, whatever they are, because their concerns may be valid. Increasing public trust in healthcare policies is most likely to happen by first inquiring about the worries of the public and then using them as a starting point for discussion (Falhquist 2018). Helps et al. (2019, 2) also emphasize that media and policy makers should avoid framing vaccination from the polarizing perspective of pro and anti-vaccination, instead recognizing the diverse nature of vaccine decisions.

If vaccine refusal works like an exit mechanism, mandatory vaccinations may end up escalating the problem instead of solving it. Mandatory vaccine policy does not necessarily increase compliance (Dare 1998) but may increase distrust (Asveld 2008: 256). According to the social science of vaccine refusal, it should be approached with great care because the increased distrust in healthcare may outweigh its potential benefits (Dubé et al. 2015: 4200). Passive compliance has been seen as a feature of the past healthcare policy, as now there is a need for a policy in which people are actively involved and respected (Vernon 2003). This is partly because the Internet has changed vaccine communication from top-down expert-consumer communication into non-hierarchical dialogue (Larson et al. 2011: 528). It has been found that vaccine refusers have a more democratic view of epistemic authority in medicine than medical authorities. Navin, especially, has argued that vaccine refusers are not necessarily irrational but often motivated by good epistemic practices such as a non-authoritarian relationship between doctors and patients (Navin 2013: 244).

Most importantly, approaching vaccine-hesitant patients with a more democratic approach undermines potential support for anti-vaccination movements (Navin 2013: 245). A shared decision-making model in healthcare can prevent people’s receptivity to anti-vaccine arguments (Chen 1999). Blume has argued that patient organizations are built on patient experiences in *not* having their grievances heard (Blume 2006: 637). Ostracized people are likely to share their grievances only with those who already share the same attitudes (Rogers & Pilgrim 1994). If the grievances are voiced only to those who already share the same attitudes, further polarization and distrust toward healthcare policy is likely to ensue. Callaghan et al. even point out that there is a pressing need to understand how racial injustices contribute to vaccine refusal, because anti-vaccine groups are framing vaccination in terms of past medical abuses against minority groups (2021: 2).

There is a need for more empirical study on the how to effectively counter vaccine refusal. Policymakers should not assume what the public wants but systematic approaches to listening public concerns should be developed (Larson et al. 2011: 532). This is because interventions that are based on empirical data and situational assessment are deemed to be more likely to succeed (Dubé et al. 2015: 4201). Dubé et al. argue that understanding the specific concerns of vaccine-hesitant individuals is necessary for designing effective strategies to counteract it. Also, the best practices in gaining back public trust after negative experiences should be better known (Blume 2006). In effect, reasoning that is driving a diminishing trust in public institutions would need to be better empirically understood (Larson et al. 2011).

How and whether people perceive herd immunity to be a public good would also need to be empirically studied (Skea et al., 2008). Then policymakers could address the public conceptions of herd immunity even if they were at odds with the medically accepted view (Skea et al. 2008). For instance, it has been found that some mothers perceive the obligation to vaccinate dependent on whether a child is especially vulnerable to the adverse effects of vaccines — whereas the official views emphasize equal responsibility to vaccinate (Skea et al. 2008).

In Hirschman’s theory, the dissatisfied are seen as a critical mass concerned with the quality of their social institutions and with a potential to change them for the better — if only their concerns were inquired, heard, and taken into consideration. The social science of vaccine refusal does not portray a picture of an irrational mass easily led by faulty argumentation that can only be brought back from hysteria by force (Rogers & Pilgrim 1994). The assumption that vaccine refusers were misinformed, and irrational has been deemed to be a sociologically inadequate explanation because this issue goes “to the heart of modern citizenship and democratic policies” (Blume 2006). Hirschman’s theory shows how people can experience alienation from the social system they inhabit instead of trying to strategically benefit from it. A lack of trust in health professionals is central in understanding vaccine refusal. A wide-spread reluctance to follow health officials’ suggestions may be a sign of exiting a public good, that is, as a collective non-acceptance of herd immunity as a public good.

## Free riding versus exit

So far, it has been argued that free riding on herd immunity is not the only, nor necessarily always the best way in bioethics to approach vaccine refusal. This is for the following reasons.

1. Free riding on herd immunity is not a significant motivating factor for vaccine refusal according to the social science studying the reasoning behind this phenomenon.
2. Vaccine refusers often spread anti-vaccination material, which would be irrational if they were only trying to benefit from the herd immunity of others. Instead, it seems that at least in some cases, they genuinely do not share the reasoning behind public vaccine programs.
3. In some cases, herd immunity has not been formed when people are refusing vaccines. There is no public good that the refusers could free ride on, nor does it necessarily get formed in the first place due to refusal. In these cases, and when (2) is met, the free riding interpretation might not be a viable way to go forward with the moral analysis of vaccine refusal as an instance of free riding on herd immunity either.
4. Interventions that social scientists suggest for vaccine refusal are not targeted to reduce the prevalence of free riding. Instead, social scientists promote utmost care in implementing mandatory policies because they may end up increasing distrust toward the healthcare system.

The exit mechanism was deemed to be an alternative way to approach vaccine refusal for the following reasons.

1. Exit mechanism explain how the public good in question does not get formed in the first place. This is because from a large enough exit from a public good, the whole existence of this good is called into question.
2. Exit correlates with a lack of trust in institutions. This is precisely what the social scientific literature on vaccine refusal suggests as one of the most significant explanatory factors in this phenomenon.
3. Exit correlates with the failure of traditional voice mechanisms such as voting. Social scientists have found partisan political preference and a lack of voting behavior to correlate with vaccine refusal.
4. Perceiving vaccine refusal as an exit mechanism correlates with communication problems between an institution and its members. This is what healthcare communication research suggests is key in understanding vaccine refusal and in designing effective interventions to counteract it.
5. The interventions suggested by Hirschman are like those suggested by social scientists studying vaccine refusal: namely listening to the refusers and trying to address their legitimate grievances.
6. Exit mechanisms explain the confusion of utilizing a traditionally market mechanism in the public arena which is also what has been deemed to be problematic by the social scientists in vaccine refusal.

Moreover, if bioethicists take free riding from herd immunity as the starting point for the ethical discussion on vaccine refusal, several morally significant factors are not taken into consideration. Individual free riding is not the only ethically suspect behavior in place when large populations refuse vaccines. Exit mechanisms consider the whole interactive situation between the organization and the individual which leads to a wide array of relevant ethical questions that have not been discussed yet. Perceiving vaccine refusal as an exit mechanism of a kind raises ethical questions beyond individual responsibility to vaccinate, such as: What is the role of healthcare institutions in making sure they do not alienate marginalized groups? What is the responsibility of an individual physician who discriminates against patients belonging to minority groups – to the extent that they become vaccine refusers?

## Conclusion

It has been argued that in ethics and the social sciences, non-normative concepts should be used when talking about what agents in society do *not* do (Kärki 2018). This is because it is difficult to discuss the normative status of an action or omission when the concepts used already invoke strong moral intuitions. Refusals in healthcare are multifaceted phenomena that require empirical interest to be fully understood (Hickson 2010). If academics decide for healthcare workers what motivates their refusals, they end up oversimplifying the issue (Hickson 2010: 180). The same goes for vaccine refusal. Without understanding actual motivations behind vaccine refusal, even though it may be possible to determine its normative status, effective interventions cannot necessarily be found. Even if free riding is a viable way to talk about vaccine refusal from the perspective of fairness, it should not be taken as an explanation of its motivation, nor as the starting point for designing effective interventions on it. A view informed by the social science of vaccine refusal leads to different interventions than those arising from a purely normative treatment. In particular, strong ascriptions of blame can backfire in actual healthcare practice.

If vaccine refusal is perceived as an instance of free riding, it is often met with sanctions. If it is perceived as stemming from lack of information, it follows that campaigns can reduce its prevalence. If it seen as an exit mechanism, addressing concerns and increasing the number and quality of feedback channels should reduce its prevalence. The social science of vaccine refusal implies consistently that respectful discussion among healthcare personnel and vaccine-hesitant individuals is key in reducing the prevalence of vaccine refusal.

How a problem is framed influences how and whether it can be solved. Refusals in general are difficult to read in healthcare practice. Bioethics can inform policy interventions on vaccine refusal, but pure normative treatment is not enough to increase understanding of this complex socio-political phenomenon that urgently needs to be tackled. A lack of neutral language for discussing a controversial issue may even hinder effective interventions by ascribing blame before understanding. To prevent further polarization, the various grievances of vaccine refusers need to be heard especially because some of them stem from legitimate worries and actual injustices experienced in healthcare practice.

## Data Availability

Not applicable.

## Footnotes

1 It must be noted that free riding on herd immunity may explain why vaccine refusal is wrong regardless of whether an agent consciously intends to free ride on herd immunity. In some cases, when herd immunity is not being formed, citizens are not adequately informed about the vaccine program, and when vaccine refusers try to convince others not to vaccinate, the moral analysis of vaccine refusal from the perspective of free riding, may also fail. However, here I argue that the emphasis on treating vaccine refusal as an instance of free riding on herd immunity, even though it does provide a useful moral analysis of the phenomenon, frames the question in a way that may limit other central ethical questions, should not be taken as an analysis of the motivations for vaccine refusal, is counterproductive at the healthcare practice, and may lead to ineffective interventions. Treating vaccine refusal as an exit mechanism not only explains its diverse motivations, but also brings different ethical questions to the fore, and provides better resources for addressing it than the kind of approaches that focus only on individual responsibility not to free ride. I must thank an anonymous reviewer for pressing these distinctions.

2 The moral analysis of vaccine refusal as free riding on herd immunity may fail when the public good of herd immunity does not get formed, when the agent does not know about the vaccine program, when vaccine refusers are trying to convince others to not vaccinate, or when other necessary features of free riding on herd immunity as a public good are not met.

3 Thank you to an anonymous reviewer for pointing this out.
